# Small and Innovative Molecules as New Strategy to Revert MDR

**DOI:** 10.3389/fonc.2014.00002

**Published:** 2014-01-21

**Authors:** Laura Zinzi, Elena Capparelli, Mariangela Cantore, Marialessandra Contino, Marcello Leopoldo, Nicola Antonio Colabufo

**Affiliations:** ^1^Dipartimento di Farmacia – Scienze del Farmaco, Università degli Studi di Bari “A. Moro”, Bari, Italy

**Keywords:** MDR, P-gp, dual effect, multitarget drugs, MDR reverting activity

## Abstract

Multidrug resistance (MDR) is a complex phenomenon principally due to the overexpression of some transmembrane proteins belonging to the ATP binding cassette (ABC) transporter family. Among these transporters, P-glycoprotein (P-gp) is mostly involved in MDR and its overexpression is the major cause of cancer therapy failure. The classical approach used to overcome MDR is the co-administration of a P-gp inhibitor and the classic antineoplastic drugs, although the results were often unsatisfactory. Different classes of P-gp ligands have been developed and, among them, Tariquidar has been extensively studied both *in vitro* and *in vivo*. Although Tariquidar has been considered for several years as the lead compound for the development of P-gp inhibitors, recent studies demonstrated it to be a substrate and inhibitor, in a dose-dependent manner. Moreover, Tariquidar structure–activity relationship studies were difficult to carry out because of the complexity of the structure that does not allow establishing the role of each moiety for P-gp activity. For this purpose, SMALL molecules bearing different scaffolds such as tetralin, biphenyl, arylthiazole, furoxane, furazan have been developed. Many of these ligands have been tested both in *in vitro* assays and in *in vivo* PET studies. These preliminary evaluations lead to obtain a library of P-gp interacting agents useful to conjugate chemotherapeutic agents displaying reduced pharmacological activity and appropriate small molecules. These molecules could get over the limits due to the antineoplastic-P-gp inhibitor co-administration since pharmacokinetic and pharmacodynamic profiles are related to a dual innovative drug.

## Introduction

Human ATP binding cassette (ABC) transporters belong to a family of 49 genes classified in seven subfamilies (A–G) (1, 2).

Some of these transporters are involved in multidrug resistance (MDR) such as ABC-B1 (P-glycoprotein, P-gp), ABC-G2 (breast cancer resistance protein, BCRP), and ABC-C1-6 (MDR associated proteins, MRP1-6) (3).

Multidrug resistance is a complex phenomenon that limits the efficacy of chemotherapeutic treatment. Some tumors are intrinsically resistant to pharmacological therapy, while others, initially sensitive to chemotherapy, become resistant during the treatment. Resistance to anticancer drugs is due to several factors such as pharmacokinetic, tumor micro-environmental changes, or cancer cell-specific factors that occur at different levels:

– increased drug efflux or decreased drug influx;
– drug inactivation;
– drug target modification;
– apoptosis evasion.

The first of these mechanisms is mediated by plasma membrane transporters such as P-gp.

Several strategies were suggested for reversing MDR and, among them, the co-administration of anticancer drugs with an ABC transporter inhibitor has been proposed to improve the bioavailability of chemotherapeutic agents (4, 5).

Among MDR pumps, P-gp is one of the most studied because of the broadest substrate specificity and the widest tissues and organs distribution such as liver, intestine, brain, and kidneys (6). This transporter actively effluxes several compounds from cells and, being overexpressed in tumor cells exerting a significant effect on the bioavailability, distribution, and activity of many drugs, especially those used in the cancer treatment (7).

**Figure 1:**
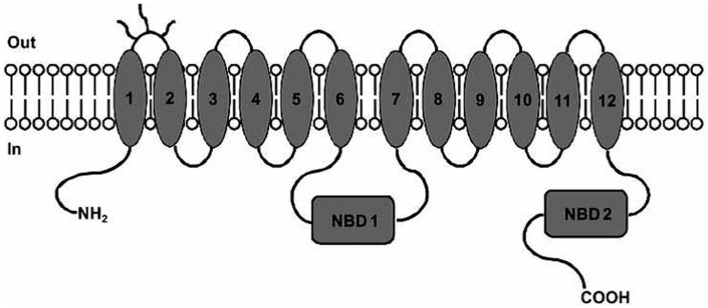
**P-gp structure: MSDs and NBDs**. Picture reported in Ref. (6).

P-glycoprotein is a 170-kDa phosphorylated glycoprotein encoded by MDR1 gene. Structurally, P-gp contains 12 transmembrane helices organized in 2 membrane spanning domains (MSDs), each containing 6 transmembrane helices and 2 nucleotide-binding domains (NBDs) responsible for ATP binding (3, 8) (Figure 1).

**Figure 2:**
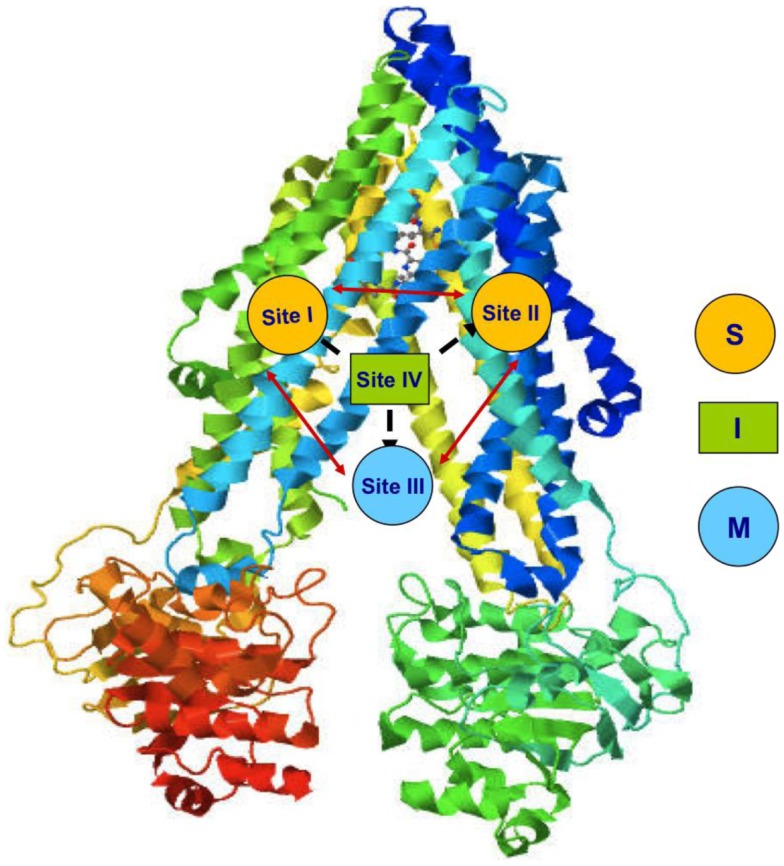
**P-gp binding sites (S: substrate, M: modulator, I: inhibitor**).

This protein uses ATP hydrolysis as the energy source for the translocation of several structurally unrelated molecules (9). This suggests the presence of different binding sites (10, 11). Indeed, four distinct interacting binding sites have been identified in P-gp structure (Figure 2). Sites I–II are assigned for the binding of substrates, site III is for the modulators, and site IV binds the inhibitors. It has been hypothesized that the binding site of inhibitors is folded to inhibit the ATP binding and so the pump, although binds the substrate, cannot extrude it. The four binding sites are able to allosterically communicate in a negative heterotropic manner and the binding to one of these sites switches the other sites to a low-affinity conformation (9).

Several models have been proposed for P-gp efflux: (1) pore, (2) flippase, (3) hydrophobic vacuum cleaner, and (4) two-cylinder engine (12, 13).

In the pore model, drugs binding P-gp to the cytosol are transported out of cells through a channel created by protein.

In the flippase model, P-gp links the drugs that are transported from the inner to the outer compartment of the plasma membrane against a concentration gradient.

In the hydrophobic vacuum cleaner model, molecules, recognized by P-gp in the lipid bilayer, enter into the protein from the membranous site and exit through the central cavity.

In the two-cylinder engine model, it has been hypothesized that P-gp contains two drug-binding sites, in which each half-transporter has its own drug carrier (14).

The translocation mechanism of P-gp was blocked by inhibitors activity (12).

**Figure 3:**
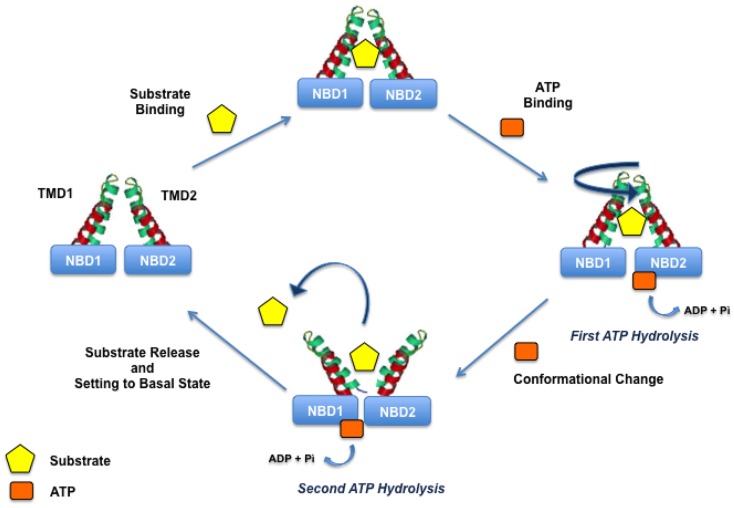
**P-gp translocation mechanism**.

Indeed, the initial step of the translocation process is the binding of drugs to an high-affinity site and simultaneously the binding of ATP to the NBDs. Drug and ATP binding are coupled to the ATP hydrolysis and two ATP molecules are needed for the turnover; the first molecule is responsible for drug translocation and the second is needed to set the transporter in the basal state (Figure 3).

**Figure 4:**
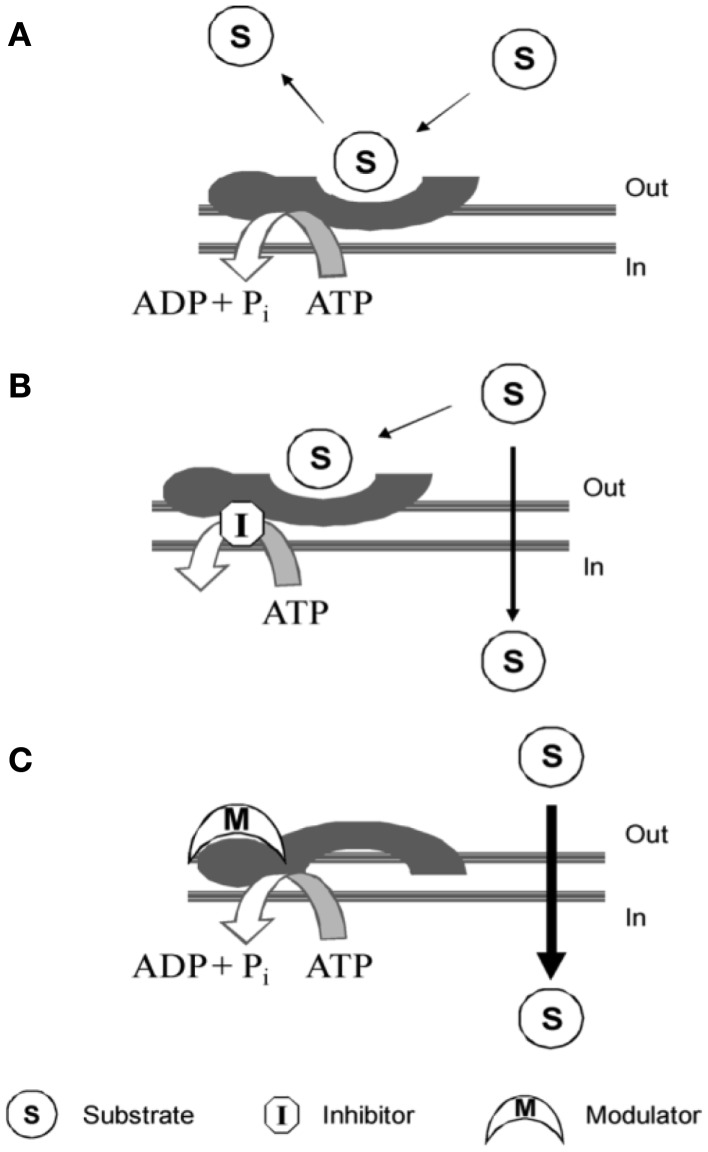
**P-gp interacting mechanism of: (A) substrate; (B) inhibitor; (C) modulator**. Picture reported in *Journal of Medicinal Chemistry* (2010) 53:1883–97.

Compounds interacting with P-gp have been classified into three categories: substrates, inhibitors, and modulators (Figure 4).

Substrates are molecules that are actively transported by the protein and therefore have a higher concentration outside the cell with respect to the cytosol (10).

High substrate concentration causes a block of the pump by saturating the substrate-binding sites and in literature, this finding led to a mistake in terms of intrinsic P-gp-interacting mechanism (10).

Modulators modify substrate-binding site through a negative allosteric mechanism. Imaging studies with radiotracers demonstrated that modulators are able to alter the substrate-binding site in a non-competitive manner, modifying the maximal receptor density (*B*_max_) but not the dissociation equilibrium constant (*K*_d_). Therefore, it suggests that allosteric communication between substrate- and modulator-binding sites exists (10).

Inhibitors block the translocation activity of P-gp by interfering with the ATP binding to NBD. However, although different mechanisms, substrates, modulators, and inhibitors could exert the same final biological effect restoring cell sensitivity to chemotherapeutic agents.

## Biological Assays

**Figure 5:**
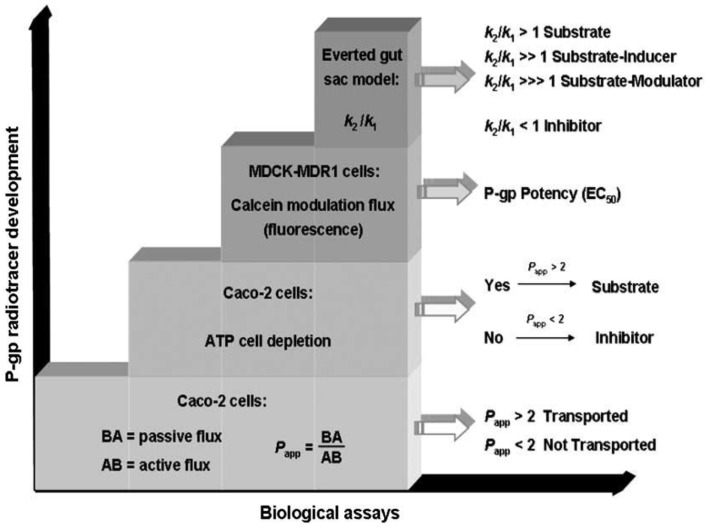
**Characterization of P-gp ligands (k_1_: influx constant; k_2_: efflux constant)**. Picture reported in Ref. (16).

The characterization of P-gp-interacting mechanism of drugs is an important task in the development of P-gp ligands and it is performed by specific biological *in vitro* assays (15) (Figure 5).

A wide range of methodologies has been used to characterize the P-gp interaction. These methods employ intact cells or purified protein and a combination of different approaches is often required to identify the mechanism of interaction.

The identification of the P-gp-interacting mechanism is performed by the combination of three biological assays:

– determination of the apparent permeability (*P*_app_);
– ATP cell depletion;
– inhibition of the P-gp-mediated transport of a fluorescent probe (Calcein or Rhodamine);
– everted gut sac model.

### Apparent permeability determination

Apparent permeability (*P*_app_) is a pharmacokinetic parameter that is determined in Caco-2 cells system, a cell monolayer model suitable for the study of the passive and active transport through the biological membranes. Indeed, in this system the Basolateral–Apical flux (B → A), representative of passive diffusion, and Apical–Basolateral flux (A → B), representative of active P-gp-modulated transport, are determined.

The BA/AB ratio is useful to identify P-gp inhibitors (BA/AB <2), P-gp substrates (BA/AB from 18 to 20), or P-gp modulators (BA/AB ranging from 2 to 18).

### ATP cell depletion

This assay, performed in Caco-2 cell monolayer and in Madin–Darby Canine Kidney cells (MDCK) overexpressing P-gp, permits to establish if the compound is able to deplete ATP. Substrates activate ATPase whereas inhibitors are not transported unchanging the ATP cell level.

### Inhibition of calcein-AM transport

This assay is useful to determine the potency (EC_50_) of P-gp ligands and is performed in MDCK cells, stably transfected for P-gp overexpression (MDCK-MDR1). The assay is carried out using a non-fluorescent prodrug, the acetoxymethyl ester of calcein (calcein-AM), which is a P-gp substrate. In the presence of a P-gp modulator, calcein-AM diffuses into the cytosol where it is hydrolyzed to the fluorescent dye calcein, that is not a P-gp substrate and since hydrophilic, it cannot diffuse through the membrane (17). In this assay also Rhodamine may be employed as a probe although calcein-AM is more useful because Rhodamine displays good cell permeability and therefore, its fluorescence determination at stationary state is more complex than calcein-AM.

### The everted gut sac model

This assay is an *ex vivo* method to study the P-gp-mediated intestinal absorption of drugs and their interactions with CYP450 enzymes (18, 19). This double information (the effect of P-gp-mediated transport and CYP450-metabolizing activity) is obtained since the everted gut sac assay is performed on isolated rat ileum where CYP450 enzymes and P-gp are present.

This combined study is needed because inhibitors and substrates may display overlapping activities toward CYP450 enzymes and the P-gp pump (20).

In this method, the transport of a known P-gp radiolabeled or fluorescent substrate, in the absence and presence of a P-gp-interacting agent, is evaluated. The flux of a P-gp substrate such as Rhodamine 125, from serosal to mucosal compartment and *vice versa*, is represented by the efflux (*k*′2) and influx rate constants (*k*′1), respectively. These determinations are carried out in the presence of a P-gp-interacting agent to determine *k*″2 and *k*″1, the efflux and influx constants of the tested substrate after P-gp interaction.

## P-gp Substrates, Modulators, Inhibitors

The most important studied P-gp ligands are classified in three different categories.

### P-gp substrates

**Chart 1:**
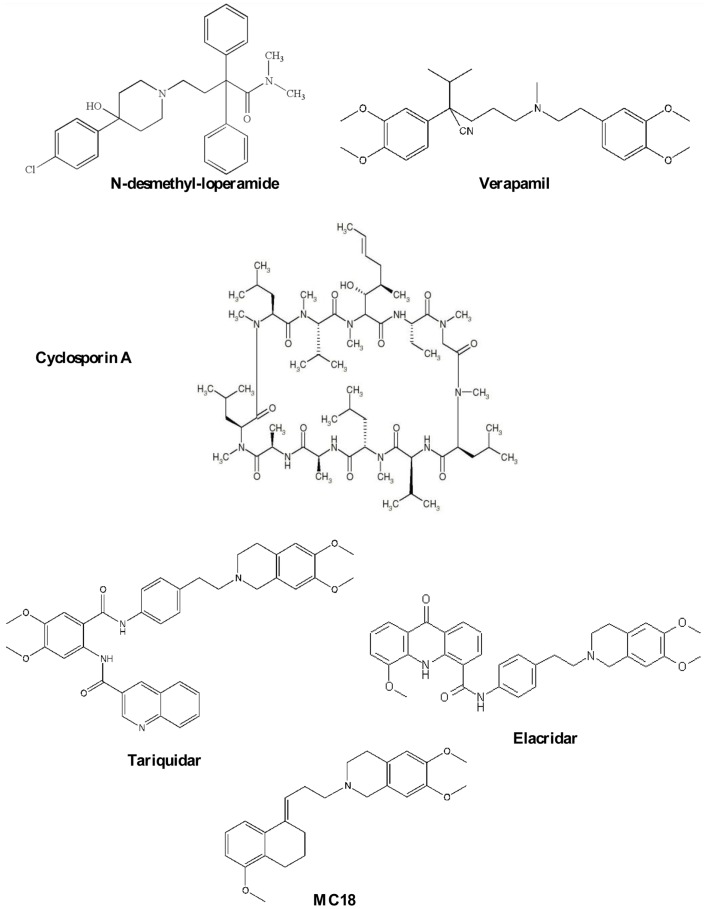
**P-gp ligands**.

This class is the most extensively studied and Verapamil and *N*-desmethyl-loperamide (Chart 1) are to date the gold standard of this class of compounds.

Verapamil, a calcium channel blocker, was found to reverse MDR (21) and it has become the reference compound for developing other P-gp substrates. It saturates the pump at high doses and therefore, it is a potential ligand for reversing MDR in co-administration with antineoplastic agent for different types of cancer (21, 22). However, verapamil cannot be employed because of toxic cardiovascular side effects. Despite this, the radiolabeled compound, ^11^C-verapamil, has been developed to visualize P-gp function and to date, it is considered to be the reference substrate for imaging P-gp activity (23). However, verapamil is quickly metabolized by CYP450 enzymes giving radiometabolites, some of which are themselves P-gp substrates (24).

*N*-desmethyl-loperamide (dLop) is the major metabolite of Loperamide and at low concentrations it acts as substrate while at high concentrations, as reported for verapamil, dLop saturates the pump (25). Also, dLop has been radiolabeled and used for imaging P-gp *in vivo* by PET analysis (26).

### P-gp modulators

Cyclosporin A (CsA, Chart 1), an immunosuppressant agent, is a P-gp modulator, widely used *in vitro* as a tool to study MDR because it restores the cell concentration of chemotherapeutic agents. In imaging studies, the co-administration of CsA with a radiolabeled P-gp substrate (27) has been performed to visualize the P-gp activity because it increases radiotracer cell uptake by modulating the P-gp-binding sites. However, CsA treatment enhances the uptake of the radioligand in all regions where P-gp is present including targeted and non-targeted tissues (2–15, 17–30).

### P-gp inhibitors

Elacridar (Chart 1) is a dual P-gp/BCRP ligand and can be orally administered. It was tested in combination with doxorubicin in patients with advanced solid tumors (31). At the recommended dose of doxorubicin, a pharmacologic hematologic toxicity was observed, mainly consisting of leukocytopenia and granulocytopenia.

Moreover, Elacridar was co-administrated with topotecan (32), a P-gp and BCRP substrate (Phase I) with unsatisfactory results (33). ^11^C-Elacridar is tested *in vivo* to evaluate the overexpression of P-gp and BCRP in human colon adenocarcinoma (33, 34).

Tariquidar (Chart 1), an anthranilic derivative, is the most potent P-gp ligand in nanomolar range. It has been co-administrated in clinical trials with chemotherapeutic agents for restoring the efficacy of therapy (35–38). Results were quite unsatisfactory because of poor selectivity against other ABC transporters that are not involved in MDR. Tariquidar has been evaluated *in vivo* for diagnosing breast tumors in animal model using (R)-^11^C-verapamil (35, 39, 40).

Recently, the suitability of ^11^C-tariquidar and ^11^C-elacridar for visualizing cerebral P-gp expression in healthy human subjects, in analogy to a previous preclinical study (16, 35), was investigated. However, ^11^C-tariquidar and ^11^C-elacridar displayed a “substrate-like *in vivo* behavior”; in particular, they are dual P-gp/BCRP substrates and these findings disagreed with *in vitro* results (41).

(*E*)-6,7-Dimethoxy-2-[3-(5-methoxy-3,4-dihydronaphthalen-1(2*H*)-ylidene)propyl]-1,2,3,4-tetrahydroisoquinoline, better known as MC18 (Chart 1), is a small molecule bearing tetralin moiety (42). To date, ^11^C-MC18 is the first P-gp inhibitor studied *in vivo* in PET studies. It displayed fourfold higher uptake in the target organs compared with ^11^C-tariquidar and ^11^C-elacridar (43).

## Strategies to Revert MDR

**Figure 6:**
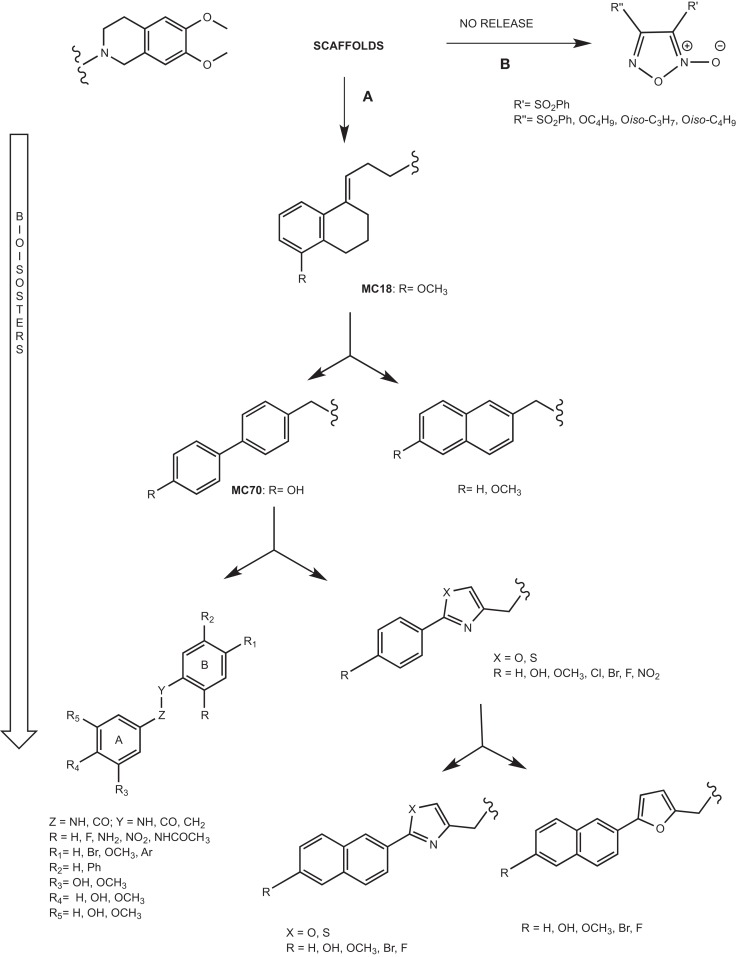
**Scaffolds of versatile libraries**. **(A)** Bioisosteric approach; **(B)** NO release ligands.

The pivotal role of P-gp in MDR has stimulated the development of P-gp ligands able to reverse the resistance to a wide number of drugs. Hence, the need to design potent and selective P-gp inhibitors stimulated the development of small molecules on which structure–activity relationship (SAR) studies could be easily and better performed. The development of these compounds is depicted in Figure 6 and it is based on the synthesis of bioisosteres obtained through subsequent lead optimization studies.

### Small libraries from versatile scaffolds

#### Tetralin derivatives

The *lead compound* of this class is MC18 (Figure 6A) (EC_50_ = 1.50 μM), bearing an (*E*)-double bond, a potent P-gp inhibitor (42). When the double bond shifts into the tetralin ring, the ligands are less potent than MC18 and are P-gp substrates. Moreover, the presence and the position of methoxy substituent on tetralin nucleus are important in terms of the potency and intrinsic activity. The saturated derivative, MC266 (EC_50_ = 6.35 μM), was the best P-gp substrate in this class. Therefore, the partial conformational restriction of spacer is involved in the P-gp-interacting mechanism. These two lead compounds, inhibitor and substrate respectively, have been ^11^C-radiolabeled and tested *in vivo* PET studies leading to significant and coherent results in comparison with the *in vitro* data previously reported (43).

#### Biphenyl and naphthyl derivatives

The conformational restriction of MC18 seems to be a requirement for improving P-gp-inhibitory activity. In order to evaluate this statement, the restriction of the spacer linking the non-basic moiety was tested in a series of molecules bearing two different fragments: 1,4-biphenyl and 2-naphthyl moieties (Figure 6A) (44).

In the biphenyl series, 4-biphenyl derivatives displayed the best activity in P-gp-inhibitory activity and among these compounds, the best result was obtained for MC70 (EC_50_ = 0.69 μM). In recent years, MC70 was extensively studied in order to confirm its P-gp-interacting activity considering its role to enhance the chemotherapeutic agent when co-administrated (45).

Although tetralin and biphenyl derivatives displayed high P-gp activity, they were active toward other ABC transporters such as BCRP and MRP1.

#### Aryloxazole and arylthiazole derivatives

**Figure 7:**
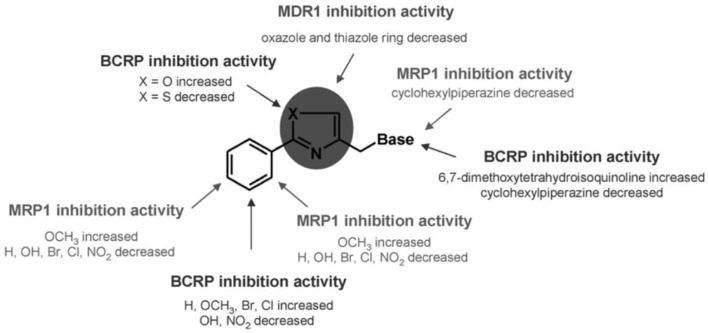
**SAR studies of aryloxazole and arylthiazole derivatives**. Picture reported in Ref. (47).

Aryloxazole and arylthiazole derivatives (Figure 6A) were designed as cycloisosters to improve the P-gp-inhibitory activity and selectivity. The results demonstrated that aryloxazole and arylthiazole derivatives, designed as cycloisosteres of biphenyl derivative MC70, were found to be less potent than the reference compound in inhibiting P-gp (46). Indeed, these compounds were screened by SAR studies toward BCRP and MRP1 giving interesting structural determinants for these pumps as depicted (Figure 7). Finally, it was found that both aryloxazoles and arylthiazoles were P-gp substrates.

Furthermore, the aryl fragments were replaced by a naphthyl nucleus and three heteronuclei (oxazole, thiazole, and furyl) have been evaluated (47). The obtained results showed that the replacement of aryl nucleus with naphthyl moiety lead to obtain compounds with three different activity profiles:

1. P-glycoprotein inhibitors: unsubstituted oxazoles or bearing −F and −OH on the naphthyl fragment;
2. Unambiguous substrates (48): oxazole bearing −OCH_3_ on the naphthyl fragment and thiazole bearing −Br and −OCH3 on the naphthyl fragment.
3. Ambiguous substrates (48): oxazole bearing Br on the naphthyl fragment and unsubstituted thiazole or bearing Br on the naphthyl fragment.

Finally, all furyl derivatives were ambiguous substrates.

#### Galloyl-based derivatives

The 3,4,5-trihydroxybenzoyl and 3,4- and 3,5-dihydroxybenzoyl fragments (Figure 6A) have been employed as scaffolds for a set of ligands that are representative of pharmacophoric nucleus of tariquidar (49).

For this purpose, compounds have been divided into four different structural series that present:

1. variation at R,
2. variation at R1,
3. polyhydroxy derivatives,
4. pyrogallol 1-methyl ethers.

The benzamides of the first set showed good P-gp-inhibitory activity (IC_50_ ranging from 20 to 1.4 μM). These outcomes led to deepen the study with two approaches:

1. keeping fixed the 3,4,5-trimethoxy-*N*-(2-nitrophenyl)benzamide scaffold and introducing a series of R1 substituents in the 4-position of the aniline moiety;
2. desmethylating one or more methoxy groups belonging to A ring.

The evaluation of data suggested that, with the exception of the 4-bromo and 4-methoxy congeners, all compounds of this second set were potent and selective P-gp inhibitors. In particular, molecules bearing R = NO_2_ and in R1 H or benzo[1,3]dioxol-5-yl displayed submicromolar activity.

The screening of the gallamide derivatives indicated a moderate inhibitory potency for P-gp, independently with respect to the number and position of phenolic groups.

In the last series, all the pyrogallol-1-monomethyl ether derivatives showed moderate P-gp-inhibitory activity.

Taking into account that the most important inhibitory activity changes were mainly concerned with the structural modifications on B ring, the role of the amide function was evaluated by testing the corresponding anilide and amine. The amine was equipotent with respect to the other tested compounds toward P-gp, despite the drastic change in terms of planarity and conformational flexibility. By contrast, the anilide showed no inhibitory activity toward P-gp. The 3,4,5-trimethoxyamide derivatives displayed moderate inhibitory activity toward P-gp.

#### Furoxan derivatives

Another strategy to reverse MDR is the nitration of a tyrosine present in TM6 domain of P-gp (50). It was reported that furoxans are able to produce *in situ* NO interacting with a thiol group. In fact, furoxans (Figure 6B) have been developed on the base of their properties to induce NO release and this pharmacological effect is the mechanism of coronary dilators (51–53). Moreover, the correlation between a decreased NO synthesis and MDR onset in doxorubicin-sensitive and doxorubicin-resistant cells has been widely reported (54). For this reason, a series of furoxan derivatives was designed and tested in activity and selectivity toward ABC transporters. Firstly, diphenylfuroxan derivatives and 3- and 4-phenylfuroxan isomer pairs, bearing different substituents with stereo-selective and lipophilic properties, were evaluated. In particular, the compounds having electron-withdrawing substituent and high lipophilic group such as phenylsulfonyl, displayed the best P-gp activity. The evaluation of phenylsulfonylfuroxan isomer pairs, bis(phenylsulfonyl)furoxan derivatives, and 3-phenylsulfonyl substituted furoxans, bearing alkoxy groups at position 4, displayed that 3-phenylsulfonyl substituted furoxans were found to be P-gp inhibitors, and the 4-substituted ligands showed the best activity and selectivity. Indeed, the best results were obtained both for 3,4-diphenylsulfonyl derivative (EC_50_ = 3.0 μM) and for alkoxy derivatives such as *n-*Butoxy, *iso-*Propoxy, and *iso-*Butoxy (EC_50_ = 2.26, 2.15, and 2.23 μM, respectively) (55).

### Dual effect drugs

Although the co-administration of a P-gp inhibitor and an antineoplastic agent could be considered a potential strategy to revert MDR, to date this approach was not clinically available because of pharmacokinetic limitations, in particular different apparent permeability, bioavailability, and metabolism. For this reason, others approaches were taken into account in the recent past:

– use of P-gp-targeted antibodies (56);
– encapsulation of anticancer drugs in liposomes (57);
– nanospheres able to circumvent MDR (58).

New innovative approaches to revert MDR could be: the development of molecules having a dual effect, potent cancer cell-killing agent and P-gp activity/expression inhibitor, and the collateral sensitivity (CS).

For example, some taxanes inhibited P-gp activity employing Rhodamine 123 as a fluorescent dye (59). These compounds could disclose new perspectives because they not only acted as cytotoxic agents but also inhibited the activity of P-gp efflux pump.

In another study (60), starting from Indirubin (a traditional Chinese medicine), the most potent derivative, PH II-7, has been evaluated. This compound showed antitumor activity inducing itself apoptosis and S phase cell cycle arrest, and in the meantime, it is not a P-gp substrate and so, high cell concentration of this compound was detected. The confocal microscopy displayed that this compound was not P-gp effluxed and significantly increased Adriamycin and Vincristine effect by reversing MDR.

### Multitarget drugs

**Figure 8:**
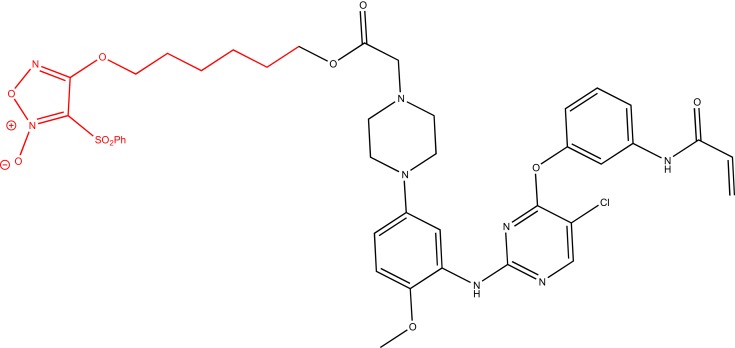
**NO-donor furoxan moiety linked to Gefitinib-like scaffold**.

Another strategy could be the design of multitarget drugs bearing scaffolds depicted in Figure 6, having antitumor and P-gp inhibitory activities. An example is the hybridization of the NO-donor furoxan scaffold with the anilinopyrimidine moiety present in Gefitinib, leading to phenylsulfonylfuroxan-anilinopyrimidine derivative (Figure 8). This compound displayed epidermal growth factor receptor (EGFR) inhibitory activity in the treatment of non-small-cell lung cancer (NSCLC) (61). It induced apoptosis in H1975 and HCC827 cells, inhibited EGFR downstream signaling in H1975 cells, and suppressed the nuclear factor-κB activation in H1975 cells. Furthermore, it released high levels of NO in H1975 cells but not in normal human cells, inducing apoptosis, inhibiting metastasis, and sensitizing tumor cells to chemotherapy by the inhibition of drug efflux transporters (51, 62). In this study, the two nuclei, furoxan and anilinopyrimidine, were separately evaluated for their effects. The activity showed by the two nuclei separately was lower than that exerted by the linked molecule. These results suggest that the antiproliferative activity of the compound might be attributed to the synergic effects of anilinopyrimidine and NO-donor moieties.

**Figure 9:**
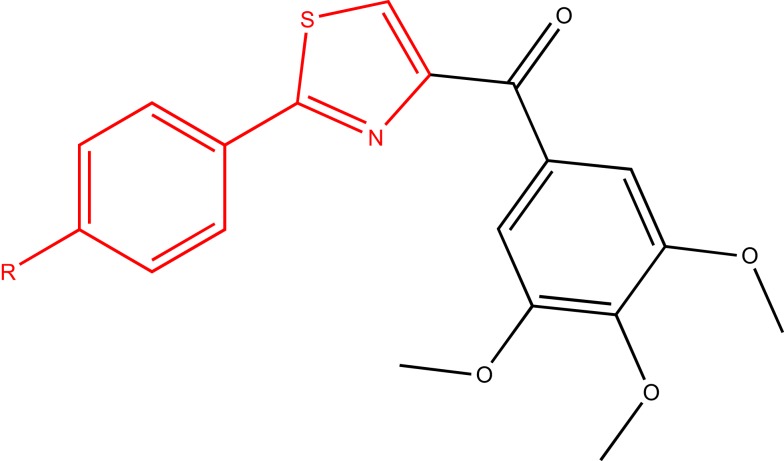
**SMART ligand**.

In another study, a class of 4-substituted methoxybenzoylaryl-thiazoles (SMART, Figure 9) was evaluated (63). These compounds exhibited great potency *in vitro* and broad spectrum cellular cytotoxicity. The *in vitro* and *in vivo* evaluation of the anticancer properties of three SMART compounds demonstrated that they potently bound to the colchicine-binding site in tubulin, inhibited tubulin polymerization, arrested cancer cells in G2/M phase, and induced apoptosis. Moreover, these compounds were able to overcome MDR since they were found equally cytotoxic in a parent cell line (OVCAR-8) and in a MDR-positive cell line (NCI/ADR-RES).

These findings demonstrated that some scaffolds such as furoxan and arylthiazole, already reported in our library as P-gp ligands, could be considered as the starting point to develop multitarget drugs. In these studies, the chemotherapeutic moiety is linked to furoxan (NO donor useful to revert MDR) or arylthiazole fragment (P-gp modulator), and the final effect was more potent that the single effect expected from each drug.

### Collateral sensitivity

An alternative potential approach to treat drug-resistant tumors is the CS where several compounds selectively kill MDR cells without affecting the non-resistant parental cells (64).

Different hypotheses have been proposed to better explain this mechanism:

– production of reactive oxygen species (ROS);
– energetic level changes;
– extrusion of essential endogenous substrates for cell survival;
– perturbation of cell membranes.

The first hypothesis takes into account that several CS agents are substrates of P-gp, stimulating ATPase activity and in the meantime, the substrate extrusion from the plasma membrane into the extracellular environment (65). Once back in the extracellular environment, substrates repeat this cycle and P-gp performs a process known as futile cycling to increase the ATP hydrolysis inducing oxidative stress. MDR cells initiate apoptosis when ROS levels overcome a certain limit. Two reported ligands inducing CS are siramesine and the P-gp substrate and σ_2_ agonist 9-[4-(6,7-dimethoxy-1,2,3,4-tetrahydroisoquinolin-2-yl)butyl]-9*H*-carbazole that generate more ROS in the MCF7/Adr than in the MCF7 cell line (66).

Another hypothesis is that P-gp-expressing cells are more sensitive to changes in energy utilization. Indeed, several compounds that interfere with cellular metabolic pathways, such as glycolysis or oxidative phosphorylation, have been identified as MDR-selective agents. The glycolysis antimetabolite 2-deoxy-d-glucose (2-DG) seems to confirm this finding. Indeed, 2-DG activates apoptosis and selectively kills numerous MDR cell lines compared to drug-sensitive parental lines.

The extrusion hypothesis asserts that CS agents mediate cytotoxicity by stimulating, sensitizing, or facilitating the extrusion of endogenous essential components. This phenomenon is not reported in P-gp-expressing cells but it may be the case for MPR1-mediated CS.

Moreover, several CS agents alter membrane biophysical properties (67). Indeed, they induce membrane perturbation in P-gp-expressing cell lines, leading to the hypothesis that changes in membrane structure and fluidity contribute to CS (68). Pentazocine and verapamil are reported to reduce membrane fluidity in the colchicine-resistant B30 cell line.

## Conclusion

To date the co-administration of a chemotherapeutic drug with a P-gp inhibitor, the encapsulation of anticancer drugs in liposomes, and the nanosphere formulation to reverse MDR failed for several reasons. This review aims to disclose new strategies in the design of multitarget drugs useful toward MDR. Interesting approaches are: (i) the development of drugs bearing a unique moiety responsible for the anticancer effect and MDR reversing activity; (ii) the design of molecules bearing different pharmacophores for multitarget activity; (iii) the evaluation of CS agents.

In the present review, we overviewed our P-gp ligands library to suggest new scaffolds that could be used to design multitarget drugs in accordance with the approaches already reported.

## Conflict of Interest Statement

The authors declare that the research was conducted in the absence of any commercial or financial relationships that could be construed as a potential conflict of interest.
